# Analysis of the Hertz Contact Model for Evaluating Mechanical Properties of Polymers Using the Finite Element Method

**DOI:** 10.3390/polym17223018

**Published:** 2025-11-13

**Authors:** Laisvidas Striska, Rokas Astrauskas, Nikolajus Kozulinas, Dainius Udris, Sonata Tolvaisiene, Eugenijus Macerauskas, Inga Morkvenaite, Arunas Ramanavicius

**Affiliations:** 1 Department of Nanotechnology, Center for Physical Sciences and Technology, Sauletekio al. 3, 10257 Vilnius, Lithuania; laisvidas.striska@ftmc.lt; 2 Department of Electrical Engineering, Vilnius Gediminas Technical University, Plytines g. 25, 10105 Vilnius, Lithuania; dainius.udris@vilniustech.lt (D.U.); sonata.tolvaisiene@vilniustech.lt (S.T.); eugenijus.macerauskas@vilniustech.lt (E.M.); 3 Faculty of Mathematics and Informatics, Vilnius University, 01513 Vilnius, Lithuania; rokas.astrauskas@mif.vu.lt (R.A.); nikolajus.kozulinas@mif.vu.lt (N.K.); 4 Department of Physical Chemistry, Vilnius University, Naugarduko g. 24, 03225 Vilnius, Lithuania

**Keywords:** atomic force microscope, elastic modulus, Young’s modulus, contact radius, finite element analysis, polymer

## Abstract

Atomic force microscopy (AFM) is widely used to quantify mechanical properties, typically Young’s modulus, by fitting force–indentation data with various mathematical contact models. However, results across laboratories often diverge, and the causes remain unresolved. Here, we interrogate the methodology by which mechanical properties are defined in AFM indentation and identify key limitations of the Hertz model, the standard model for determining mechanical properties, notably that the contact radius is not directly determined, which limits the accuracy of the estimated Young’s modulus. We hypothesize that this inference systematically overestimates the true tip–sample contact, which inflates the contact area and thereby underestimates Young’s modulus. This bias is amplified under large indentation conditions, which are frequently used in soft-material studies. To isolate and clarify the issue, we focus on a well-characterized polymer, polyvinyl chloride (PVC), using it as a controlled testbed for contact radius overestimation. Our analysis is focused on the contact radius and Hertz-based extraction of Young’s modulus. We determined the contact radius and Young’s modulus using AFM with two different probes: a sphere with a 20 nm radius (SPHERE20) and a sphere with a 2 µm radius (SPHERE2000). The results were compared to macroscopic data obtained using a standard measurement (ISO 527-1:2019) of Young’s modulus. The contact was modeled using finite element analysis (FEA). The dependence of the contact radius on the indentation was compared to the Hertz model. The results from FEA fit corrected contact radius values, and it is smaller by 15.46% (SPHERE20) and 57.9% (SPHERE2000) than those calculated by the Hertz model.

## 1. Introduction

Atomic force microscopy (AFM) is widely used to quantify the material mechanical properties from force–indentation curves using contact models, most commonly Hertzian formulations [1,2,3]. The Hertz model allows for finding the elastic modulus from force–distance curves directly. Commercial AFM software typically ships with Hertz fits as the default method to extract the modulus [4]. Although the Hertz model persists as the standard analytical tool for AFM indentation, its core assumptions often fail for polymeric or soft materials, including living cells. Specifically, this model cannot directly determine the real contact radius, which leads to an underestimation of the Young‘s modulus [5,6]. Moreover, neither the exact tip–sample contact point nor the true indentation depth is directly observed in the Hertz model [6]; both are reconstructed from piezo motion and cantilever deflection and are only well-defined on nominally hard, smooth surfaces [5]. For mechanically soft (low Young’s modulus) samples or at large indentations, the measured deflection–piezo displacement relation becomes nonlinear, so treating it as linear biases force and indentation, compromising Young’s modulus fits [7,8]. Additional complexities, such as load-dependent contact area, surface roughness, adhesion, and finite thickness, further complicate the estimation of the Young’s modulus [9,10,11,12]. Applying a single, idealized contact model across heterogeneous tip geometries (spherical, conical, flat-ended) and adhesion models (e.g., JKR/Maugis) cannot evaluate Young’s modulus [13].

These problems result in differences in Young‘s modulus for identical materials between laboratories [5]. It has important implications for the reliability of nanoscale mechanical properties defined across materials [14]. Finite element analysis is a valuable tool for addressing this issue, as it enables the visualization and quantification of the actual contact geometry during indentation. This allows for a more accurate description of what is happening at the contact point during the indentation process.

The contact radius is a primary, often overlooked factor that causes uncertainty in the determination of Young’s modulus. We hypothesize that the real contact radius is overestimated, especially under large indentations commonly used in soft-matter studies, where the apparent contact area is higher than expected. Therefore, the real Young’s modulus is also underestimated. In the Hertz model, contact radius is not a directly measured quantity [6]; instead, it is calculated backward from the indentation depth and the assumed tip geometry. Moreover, the contact area increases with indentation [15,16]. Therefore, it is unclear what part of the results depends on the geometry of the tip and what part depends on the sample properties.

Polymer tests are beneficial as a model for soft biological materials, where large indentations are often necessary [17,18]. Polymers are easy to handle, have well-characterized Young’s moduli, and yield reproducible measurements, enabling trustworthy comparisons across experiments [19,20]. They also allowed us to isolate a key Hertz limitation: the need to determine the actual contact radius rather than relying on geometric estimates. By clarifying this behavior in samples with known properties, we can project its impact on soft tissues and motivate deeper investigation with finite element analysis. We can then transfer the improved understanding to mechanical property determination in biological materials.

The present paper aims to demonstrate the limitations of the Hertz model for AFM nanoindentation arising from indirect (and inflated) contact radius. We focused exclusively on the Hertz model, treating it as the community’s default benchmark while examining how contact radius assumptions influence modulus estimates. Additionally, we investigate how AFM indentation determines mechanical properties and identify limitations that arise specifically within the Hertz framework. We determined the contact radius and Young’s modulus using AFM with two different probes: a sphere with a 20 nm radius (SPHERE20) and a sphere with a 2 µm radius (SPHERE2000). The results were compared to macroscopic data obtained using a standard measurement (ISO 527-1:2019) [21] of Young’s modulus. Finite element analysis was performed using COMSOL Multiphysics 6.3 to analyze the mechanical contact between a rigid spherical indenter and a polymer surface during AFM indentation.

## 2. Materials and Methods

### 2.1. Materials and Equipment

The computer-controlled tension–compression test system, Mecmesin MultiTest 2.5-I (Mecmesin Limited, West Sussex, UK), with a load sensor measurement error of 0.1%, was used for stress and strain measurements. The testing machine was controlled using the Emperor Force software (Mecmesin Ltd., Horsham, UK).

**Figure 1:**
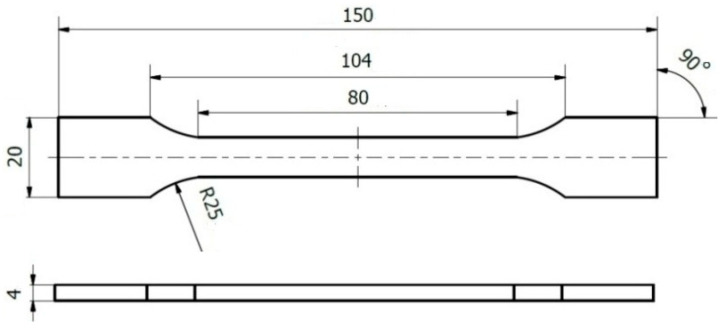
Specimen dimensions and application of the standard method for the evaluation of elastic modulus E.

Polyvinyl chloride (PVC) sheets were obtained from Heliopolis (Vilnius, Lithuania). Specimens were virgin, unfilled general-purpose grades without additional compounding; sheets were uncoated and free of prior loading or deformation to avoid bias in measured mechanical properties. Sheets were conditioned at 23 °C for at least 24 h before testing. Specimens were prepared according to the geometry shown in Figure 1 as per the ISO 527-1:2019 standard.

The elastic modulus was determined from the initial stress–strain curves (Figure 1) and fitted using [22]:

σ = E_a_·ε_n_ (1)

where E_a_ is the approximated modulus of elasticity, ε is the relative deformation, and n is the coefficient indicating the curve type (n = 1 corresponds to a linear curve consistent with Hooke’s Law, i.e., the conditional elastic limit for polymers). The specimens’ results were approximated specifically within this region.

We used the BioScope II AFM and an optical microscope, both developed by Veeco Instruments Ltd. (Santa Barbara, CA, USA), to measure force–indentation curves. Spring constant calibration of the AFM cantilevers was performed using the Thermal Tune method with the Bruker BioScope II/NanoScope V system under ambient (air) conditions [23].

### 2.2. Determination of Young’s Modulus and Contact Radius

The force–indentation curves were measured by AFM using two probes of distinct geometry (Table 1). Each cantilever has a nominal, minimal, and maximum tip radius, which significantly influences the contact radius and the final mechanical properties of the specimen. In this paper, we used only nominal tip radii. Each measurement was performed at least 25 times. The reference values of Young’s modulus were determined using the standard method (Section 2.1). The AFM/Hertz values of Young’s modulus were calculated by fitting Equation (2) to the measured force–indentation dependencies, with nominal tip radius values (Table 1).

The corrected contact radius was calculated using the tip radius obtained by fitting the Hertz model (Equation (3)) with reference values of Young’s modulus. We followed the model that AFM manufacturers themselves present as the standard model for AFM force–distance curve analysis [4].

AFM/Hertz values of the contact radius were calculated using the nominal tip radius (Table 1). The indentation values for the corrected and AFM/Hertz values were taken from experimental data.

The Young’s modulus *E* was calculated by fitting the Hertz model [24]:

(2) $$F=\frac{4}{3}\frac{E}{\left(1-\upsilon^{2}\right)}\sqrt{R}\delta^{3/2}$$

where *F* is the indentation force, *E* is Young’s modulus, *ν* is Poisson’s ratio, *R* is the radius of the tip, and *δ* is the indentation.

Contact radius was calculated [25]:

(3) $$a=\sqrt{\delta \cdot R}$$

### 2.3. Modeling and Simulation

Finite element models were developed to analyze the indentation of a spherical tip into a PVC sheet during AFM experiments. The stationary axisymmetric model in COMSOL Multiphysics was utilized (Figure 1). The indenter was modeled as a rigid material consisting of silicon nitride with a prescribed displacement along the *z*-axis. The polymer was introduced as a hyperelastic material utilizing the St. Venant–Kirchhoff model with Young’s modulus 1.15 GPa, Poisson’s ratio 0.32. The thickness of the PVC sheet is 2 mm, and the width is sufficiently large. The interaction between the tip and the polymer surface was modeled by the Augmented Lagrangian contact method. The contact was assumed to be frictionless. A roller constraint was applied to the bottom boundary, ensuring that displacement in the *z* direction is zero.

**Figure 2:**
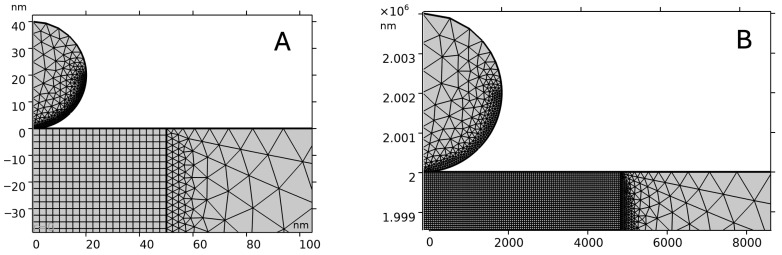
FEA model: mesh distribution. (**A**) SPHERE20, (**B**) SPHERE2000.

The model was meshed using triangular elements for the tip. Two different meshes were applied to the polymer sheet: quadrilateral elements were used in the domain below the indenter, and triangular elements were used for the remaining domain of the polymer. The computation showed that such a connected mesh improved the convergence of the model. The density of the mesh was increased near the contact area by specifying a denser distribution of mesh points (Figure 2).

The simulations provided predictions of the contact radius, deformation field, and force–indentation behavior, allowing for direct comparison with Hertzian analytical predictions and AFM experimental data.

The finite element model was further evaluated using the experimental AFM indentation data for both SPHERE20 and SPHERE2000 probes. Validation was performed by directly comparing the simulated and experimental force–indentation curves under identical geometric and material conditions. The same indentation depths and tip radii were used in the simulations as in the corresponding AFM measurements.

## 3. Results

### 3.1. Specimens’ Young’s Modulus Determination by the Standard Method

**Figure 3:**
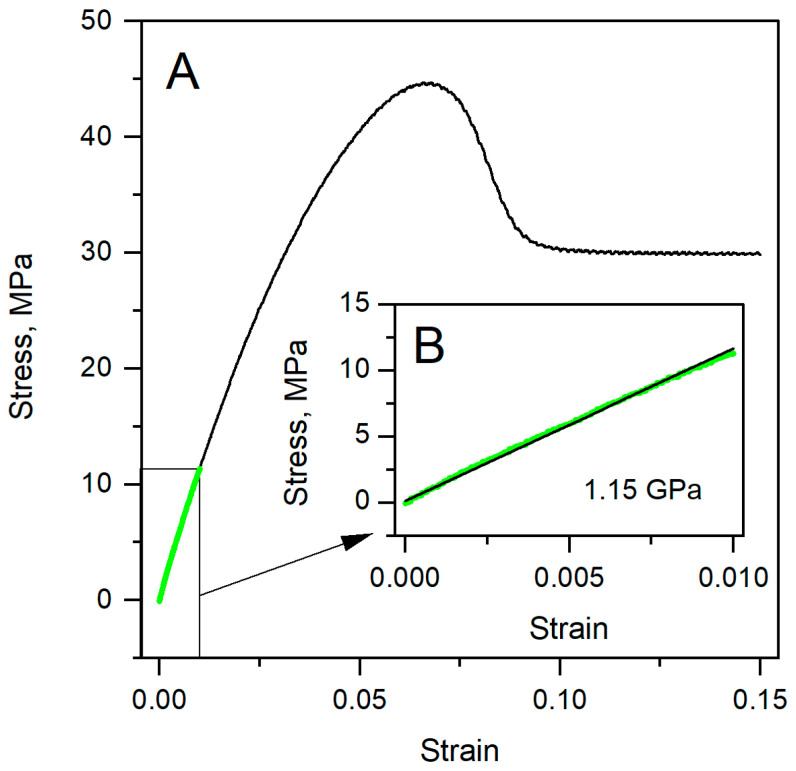
Stress–strain dependencies. (**A**) Measured PVC strain–stress dependence by the standard (ISO 527-1:2019) method. (**B**) Fitted data from A part in the elastic deformation region by Equation (1).

The stress–strain relationships of PVC were obtained using the standard tensile test according to ISO 527-1:2019. The Young’s modulus for PVC was determined from the slope of the stress–strain curve, as defined by Equation (1). The approximate reference value was 1.15 GPa for PVC. Figure 3A shows a stress–strain curve, and Figure 3B zooms in on the initial linear segment used to extract Young’s modulus. We fit only the elastic region (the boxed segment in Figure 3A) because this is where stress is proportional to strain, and the ISO tensile method defines *E*. Beyond this region, the behavior of these polymers was not analyzed for modulus determination [22].

Staying within the elastic segment minimizes nonlinear effects and avoids artifacts from plasticity, ensuring that the slope truly reflects the Young’s modulus. These ISO-style tensile moduli serve as our approved reference values for comparison with the modulus received from the Hertz model (Table 2 and Table 3).

### 3.2. Validation of the Finite-Element Model

**Figure 4:**
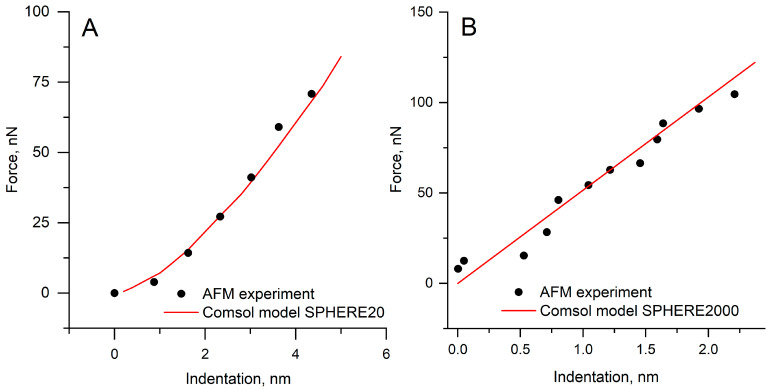
FEA model comparison with experimental data. (**A**). SPHERE20; (**B**). SPHERE2000.

Figure 4 presents a direct comparison between the finite element simulations and the experimental AFM indentation curves obtained for the SPHERE20 and SPHERE2000 probes. The numerical and experimental curves overlap across nearly the entire indentation range, indicating that the model accurately shows small-radius contact. The maximum difference in load at the comparable indentation depths does not exceed approximately 5%, which lies within the experimental uncertainty. The good agreement between the simulated and experimental curves confirmed that the finite element configuration accurately represents the contact mechanics of the system and provides a reliable basis for determining the contact radius.

### 3.3. Specimens Determination of Young’s Modulus by the Hertz Model

**Figure 5:**
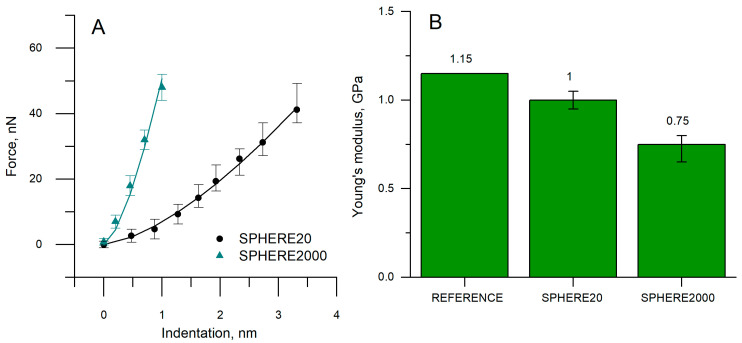
(**A**) Force vs. indentation curves and (**B**) Young’s modulus values in different cantilevers.

Figure 5 shows the force–indentation curves obtained for PVC with two different cantilevers and Young’s modulus. Here we compare the reference modulus values of PVC (1.15 GPa) with values extracted from AFM force–indentation curves using the Hertz model for two cantilever probes: SPHERE20 and SPHERE2000 (Figure 5A). The AFM/Hertz modulus underestimates the reference by ~13% for SPHERE20 (1.00 GPa) and ~35% for SPHERE2000 (0.75 GPa) (Table 2). Thus, across the two configurations, the bias spans 13–35%, with the best agreement for SPHERE20 on PVC. As discussed earlier, the probe of larger radius (SPHERE2000) overestimates the contact radius, which results in a low Young’s modulus obtained by fitting the Hertz model. The small sphere contact radius stays closer to Hertz’s assumptions and fits better.

Both probes reach comparable peak force, but for any given force, the SPHERE2000 requires much smaller indentation than the SPHERE20 (Figure 5A). Despite this logical behavior, the fitted moduli are not comparable between probes. From the same PVC material and test conditions, the Hertz model results in a difference in Young’s moduli by ~25%, and neither value reaches the ISO tensile reference of 1.15 GPa (Figure 5B).

Even though we tested a well-characterized polymer (not soft materials, like living cells), we still observed large deviations of Young’s modulus values between the probes. In principle, the Hertz model should return the same modulus for the same material once the correct tip radius R is used in calculations; in practice, this is not enough. Probe-dependent overestimation of the real, pressure-bearing contact area makes the modulus probe-specific, and both estimates fall short of the reference Young’s modulus. This aligns with reports of discrepancies in Young’s modulus between laboratories, especially for biological tissues and living cells, and underscores the need to determine or correct the real contact area when reporting AFM-derived moduli.

The value of Young’s modulus, using the corrected contact radius (Table 2), was obtained by fitting the Hertz model. We adjusted the contact radius until the Young’s modulus equals the reference value; the post-correction Δ is therefore 0.

**Figure 6:**
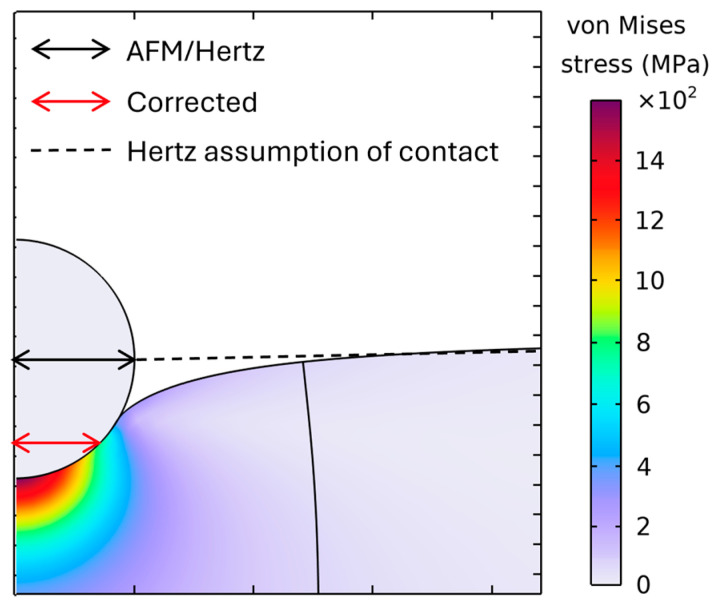
Visualization of contact radius: Hertz assumption vs. FEA.

The observed Δ indicates that the contact radius is overestimated when the nominal tip geometry is used to fit the Hertz model, which cannot directly determine the contact radius. Part of the measured indentation arises from a surrounding, non-pressurized deformation zone outside the actual contact patch (Figure 6). Consequently, the real contact radius is smaller than the Hertz model estimate, which biases the contact radius high. This inflated contact radius results in a lower fitted modulus than the reference value.

Figure 6 shows a finite element analysis of a spherical indenter on a PVC specimen. The contact patch is the high-stress zone at the interface. The dashed line marks the Hertz-assumed contact boundary, while the black arrows indicate the AFM/Hertz inferred radius, and the red arrow shows the corrected (FEA) radius. The observed FEA patch is smaller than the Hertz/geometric estimate, revealing a systematic overestimation of the contact radius. Additionally, the FEA revealed a surrounding non-pressurized deformation zone, which is not evaluated in the Hertz model. An inflated contact radius results in a lower fitted modulus from experimental force–indentation curves. In the Hertz model, which is applied for the calculation of Young’s modulus from force–indentation curves, the contact radius is not known, and only the tip radius is evaluated (Equation (2)).

### 3.4. Contact Radius Determined by Hertz Model

**Figure 7:**
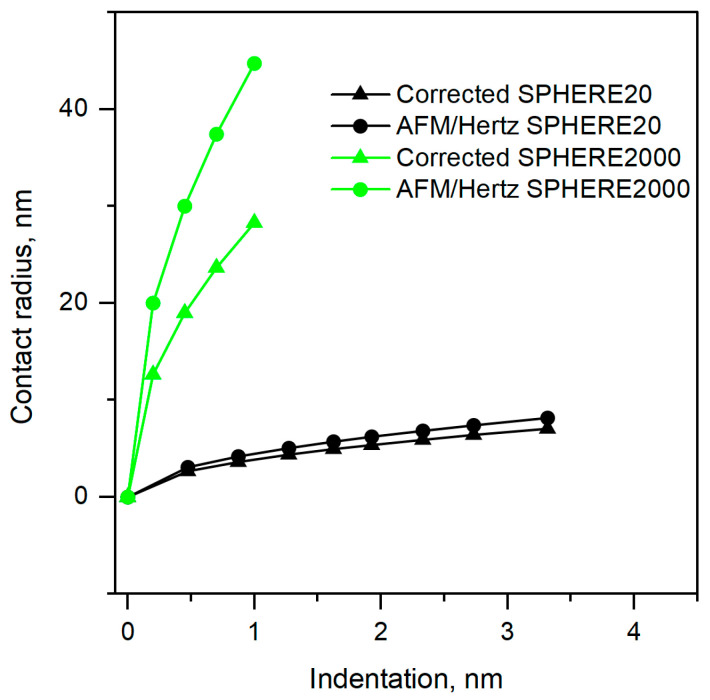
Contact radius comparison: Hertz assumption vs. corrected by Equation (3), with Young’s modulus reference from Table 2.

The contact radii were calculated by Equation (3) with the Young’s modulus values provided in Table 2. The sensitivity to indentation depth and tip geometry follows directly from contact mechanics: spherical indenters with a large radius cause a broader non-pressurized surface deformation. At the same time, the Hertz model effectively attributes to the nominal contact area, thereby biasing the fit. Accordingly, errors increase with indentation depth (Figure 7) and are greatest for SPHERE2000, which has the largest effective radii among the probes examined.

For SPHERE20, the corrected radius is 7.05 nm, and the AFM/Hertz radius is 8.14 nm, i.e., +15.5% overestimation (Table 3). For SPHERE2000, the corrected radius is 28.3 nm versus 44.7 nm from AFM/Hertz, i.e., +57.9% overestimation. Thus, the better agreement is obtained with SPHERE20 (smallest bias), whereas SPHERE2000, with the large tip radius, folds non-pressurized deformation into the depth and inflates the apparent contact area (Figure 6).

When the contact radius is calculated using the reference Young‘s modulus (Equation (3)), the corrected contact radius is consistently smaller than the AFM/Hertz value. The discrepancy increases with indenter size: the mean overestimation is approximately 15.46% for SPHERE20 and 57.9% for SPHERE2000.

In biological tissues, by contrast, larger indenters and greater depths are often required to estimate the overall Young’s modulus. In contrast, small indenters emphasize local structures, such as cell membranes, and thus yield locally biased modulus values.

Because Hertz overestimates the contact radius, the fitted Young’s modulus is lower than the reference modulus, because a larger assumed contact radius requires a smaller modulus to fit the same force–indentation curve data.

Figure 7 summarizes the contact radius versus indentation behavior, comparing AFM/Hertz estimates with corrected values for SPHERE20 and SPHERE2000. The SPHERE20 curves, both corrected and AFM/Hertz, nearly coincide across the full range: contact radius grows slowly with indentation, and the Hertz estimate sits only slightly above the corrected curve. By contrast, the SPHERE2000 curves separate strongly: for the same indentation, the AFM/Hertz radius is much larger than the corrected one, and the gap widens with depth, indicating systematic overestimation. At the representative comparison point from Table 3, SPHERE20 shows only +15% overestimation of contact radius, whereas SPHERE2000 shows +58%. Thus, SPHERE20 provides better agreement with the Hertz model, while SPHERE2000 shows a large and depth-dependent bias.

When the contact radius is calculated from the reference Young‘s modulus (Equation (3)), the corrected contact radius is consistently smaller than the AFM/Hertz value. The discrepancy increases with indenter size. Because Hertz overestimates the contact radius, the fitted Young’s modulus is lower than the reference modulus in Table 2, because a larger assumed contact radius requires a smaller modulus to fit the same force–indentation curve data.

**Figure 8:**
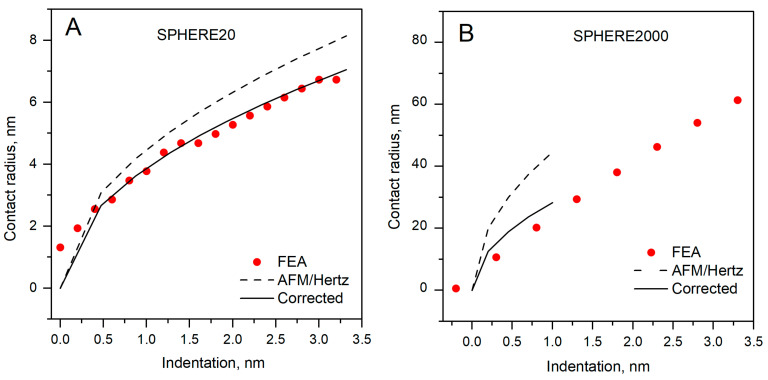
Contact radius dependence on indentation. (**A**) SPHERE20; (**B**) SPHERE2000.

The contact radius as a function of indentation is shown in Figure 8. The Hertz curve lies above the corrected and FEA curves, showing systematic overestimation of the contact radius when Hertz is used directly. The effect increases with indentation and is much larger for SPHERE2000; for SPHERE20, the separation is modest, consistent with our earlier presented errors and the closer modulus agreement with the Hertz model for the small sphere.

The FEA closely follows the corrected curve for both probes, indicating that the model captures the pressure-bearing contact patch and is suitable for further study of probe–specimen contact definitions. This supports our main conclusion: the overestimated contact area explains the low bias in the AFM-derived modulus and the lack of comparability of the obtained modulus between probes, even on the same material.

## 4. Discussion

The results of this study demonstrate a systematic discrepancy between mechanical properties obtained from AFM indentation analyzed using the Hertz model and those measured by the reference method. Specifically, the contact radius determined from the Hertz model is consistently overestimated when compared to finite element analysis (FEA) and corrected values derived from ISO-standard tensile data. This overestimation—15.5% for the SPHERE20 probe and 57.9% for SPHERE2000—leads directly to the underestimation of the Young’s modulus by 13% (SPHERE20) and 35% (SPHERE2000). Such systematic bias reflects a fundamental limitation of the Hertz model when applied to polymeric or soft materials, where the real contact radius cannot be directly measured.

The observed differences between SPHERE20 and SPHERE2000 can be attributed to their distinct contact geometries. The larger probe (SPHERE2000) produces a wider deformation zone, which includes a significant non-pressurized region surrounding the real contact patch. The Hertz model, however, assumes uniform stress distribution across the entire geometrically defined contact area. As a result, the apparent contact radius is inflated, and the corresponding fitted modulus is reduced. Similar depth-dependent artifacts have been reported in earlier nanoindentation studies, which found that indentation depth and tip curvature strongly influence measured moduli of polymers and biological materials [5,26]. The present study provides validation of this phenomenon through FEA, which accurately visualizes the real contact radius.

The FEA further revealed that the deformation field under the tip is not confined to the theoretical contact patch predicted by Hertzian theory. This explains why the contact area calculated from indentation depth alone is larger than the actual contact zone.

The better agreement obtained with the smaller SPHERE20 probe (13% deviation) supports the idea that the Hertz model remains valid within a limited range of contact radii and indentation depths. Under these conditions, the strain field is more localized, and the deformation behavior more closely approximates linear elasticity. In contrast, the SPHERE2000 probe produces a greater real contact radius mismatch with the Hertz model. These findings align with recent studies reporting probe-size dependence of AFM-derived moduli in polymers and biological cells [27,28,29].

Our findings also have implications beyond polymer testing. Biological samples, including cells and tissues, are routinely characterized by AFM under conditions where Hertzian assumptions break down [30,31]. Since such samples are measured with larger indentations, the overestimation of contact radius can be even more pronounced than in polymers, leading to severe underestimation of modulus. This may partially explain why reported cell moduli vary widely under similar experimental conditions.

The current approach provides the foundation for future extensions that incorporate viscoelasticity, adhesion, and friction effects into FEA simulations. Modeling these parameters will further enhance the reliability of modulus calculation and enable more accurate interpretation of AFM data in soft materials and biological contexts.

Table 4 summarizes representative AFM and nanoindentation studies performed on polymers. In nearly all cases, deviations from the Hertzian prediction were observed when indentation exceeded the small-strain regime or when the contact radius was comparable to the film thickness. The present work extends these comparisons by providing FEA validation of the real contact radius for spherical probes (SPHERE20 and SPHERE2000).

## 5. Conclusions

This study demonstrated that the Hertz model systematically overestimates the contact radius during AFM indentation of polymeric materials, leading to an underestimation of the actual Young’s modulus. Finite element analysis (FEA) confirmed that the real contact area is smaller than the geometrical contact area assumed by the Hertz model. For the SPHERE20 probe, the contact radius was overestimated by 15.5%, while for SPHERE2000, the overestimation reached 57.9%. Consequently, the Young’s modulus values derived from the Hertz model were 13–35% lower than those obtained by tensile testing. The reference modulus of PVC was 1.15 GPa, compared to the AFM-Hertz-derived values of 1 GPa (SPHERE20) and 0.75 GPa (SPHERE2000). These results demonstrate that geometric effects dominate the observed deviations and represent the principal limitation of Hertzian analysis in polymer indentation.

The findings clarify the source of variability often observed in AFM-based mechanical measurements of soft materials. Differences in probe radius produce systematic contact radius bias, which affects modulus determination. By integrating FEA with AFM indentation, an accurate estimation of the real contact radius is provided. This combined experimental–numerical strategy improves the reliability and reproducibility of nanomechanical characterization of polymers.

The methodology developed here establishes a foundation for probe-specific correction factors that can be integrated into standard AFM analysis workflows. Future work will expand the finite element framework to include viscoelastic, adhesive, and frictional effects to better represent realistic deformation conditions in polymers and biological materials. Incorporating such corrections into commercial AFM software could significantly enhance the comparability and standardization of nanoscale mechanical testing across laboratories.

## Figures and Tables

**Table 1:** Characteristics of cantilevers.

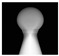	SPHERE20: HDCT (biosphere B20-NCH). Geometry: Spherical. Dimensions: T = 4 µm, L = 125 µm, W = 30 µm, f_0_ = 330 kHz. Spring constant: 40 N/m. Measured: 52.5 N/m. Tip radius: 20 nm ± 5 nm. Nominal tip radius: 20 nm. Nanotools (Munchen, Germany).
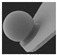	SPHERE2000: CP-FM-SiO-A-5. Geometry: Spherical. Dimensions: T = 3 ± 1 µm, L = 225 ± 10 µm, W = 28 ± 7.5 µm. Spring constant: 0.5–9.5 N/m. Measured: 4.18 N/m. f0 = 45–115 kHz. Tip radius: 2 µm ± 5%. Nominal tip radius: 2 µm. sQube (Sofia, Bulgaria).

**Table 2:** Young’s modulus, AFM/Hertz vs. reference value.

Tip	Reference ISO [GPa]	AFM/Hertz [GPa]	Δ (GPa)	Δ (%)	With Corrected Contact Radius (GPa)	Δ with Corrected Contact Radius (GPa)
SPHERE20	PVC (1.15)	1.00	−0.15	−13.00	1.15	0.00
SPHERE2000		0.75	−0.40	−35.00	1.15	0.00

**Table 3:** Contact radius at highest indentation (Figure 4): corrected vs. AFM/Hertz.

Tip	Corrected, nm	AFM/Hertz, nm	Δa	a Overestimation [%]
SPHERE20	7.05	8.14	1.09	15.46
SPHERE2000	28.3	44.7	16.4	57.9

**Table 4:** Comparison of previous AFM indentation studies on polymers.

Polymer Material	Probe Used	Analyzed Property	Model Used	Findings and Deviation	Ref.
Various polymers	AFM and FEA combined	Viscoelastic modulus	Hertz + time-dependent model	Time-dependent modulus variations up to 40%; relaxation significant	[20]
PMMA, PS	AFM spherical probe (100–500 nm)	Young’s modulus	Hertz + FEA correction	Demonstrated tip–radius dependence of modulus up to 20%; smaller probes yield higher apparent stiffness.	[19]
Epoxy and PET blends	PeakForce QNM	Modulus mapping	DMT and Hertz	Reported nanoscale modulus variation depending on probe curvature and phase heterogeneity.	[2]
Soft polyurethane	AFM colloidal (2 µm)	Viscoelastic modulus	Hertz + time-dependent correction	Introduced rate-dependent model; large probes underestimated modulus due to creep.	[5]
Poly(lactic acid) composites	AFM tip (50–200 nm)	Elastic modulus	Hertz model	Observed probe–size dependence: smaller tips gave up to 30% higher modulus values.	[7]
PMMA thin films	AFM spherical tip (500 nm)	Contact radius and modulus	Hertz + FEA validation	Finite element correction reduced Hertz overestimation of contact radius by ~25%.	[29]
Soft biological-like polymers	Spherical tip (2–10 µm)	Elastic modulus	Hertz vs. Hyperelastic model	Showed breakdown of Hertz assumptions under large deformations; hyperelastic model improved fit.	[30]
PVC	Spherical AFM tips, with radius 20 nm and 2000 nm	Hertz contact radius vs. real (corrected)	Hertz and FEA	Contact radius was overestimated by 15.5% and 57.9%	Present work

PMMA—poly(methyl methacrylate); PS—polystyrene; PDMS—polydimethylsiloxane; PEG—polyethylene glycol; PLA—poly(lactic acid); FEA—finite element analysis; AFM—atomic force microscopy; QNM—quantitative nanomechanical mapping; JKR—Johnson–Kendall–Roberts model; DMT—Derjaguin–Muller–Toporov model.

## Data Availability

The original contributions presented in this study are included in the article material. Further inquiries can be directed to the corresponding authors.
